# Implementing community-based interventions for the management of chronic conditions in low- and middle-income countries: A scoping review of qualitative evidence

**DOI:** 10.1371/journal.pgph.0004860

**Published:** 2025-07-08

**Authors:** Syreen Hassan, Nancy Kagwanja, Brahima Diallo, Robinson Oyando, Pablo Perel, Anthony Etyang, Benjamin Tsofa, Ellen Nolte, IHCoR-Africa Collaborators

**Affiliations:** 1 Department of Noncommunicable Disease Epidemiology, Faculty of Epidemiology and Population Health, London School of Hygiene and Tropical Medicine, London, United Kingdom; 2 Department of Health Systems and Research Ethics, Kenya Medical Research Institute Wellcome Trust Research Programme (KEMRI-WTRP), Kilifi, Kenya; 3 Department of Nutrition and Planetary Health, Medical Research Council Unit The Gambia (MRCG) at London School of Hygiene and Tropical Medicine, Banjul, The Gambia; 4 Health Economics Research Unit, KEMRI Wellcome Trust Research Programme, Nairobi, Kenya; 5 Department of Epidemiology and Demography, KEMRI Wellcome Trust Research Programme, Kilifi, Kenya; 6 Department of Health Services Research and Policy, Faculty of Public Health and Policy, London School of Hygiene and Tropical Medicine, London, United Kingdom; PLOS: Public Library of Science, UNITED STATES OF AMERICA

## Abstract

The rising prevalence of chronic diseases in low- and middle-income countries (LMICs) poses significant challenges to already overburdened health systems. Community-based interventions are recognised as effective strategies for managing these conditions. However, implementing such interventions faces barriers that can hinder their effectiveness. This scoping review aims to assess qualitative studies examining barriers and facilitators to implementing community-based interventions for chronic disease management in LMICs. We searched six databases for studies published between 2013–2024. Eligible studies were those with a qualitative design that explored implementation challenges and facilitators of community-based interventions. Data were thematically analysed and interpreted using the Socio-Ecological Model (SEM) to capture multi-level influences on implementation. Eighteen studies were included, covering interventions in 13 LMICs. We identified four levels of influencing the implementation of chronic condition management interventions: individual (service users and providers), community, health system/policy, and interpersonal. Barriers at the individual level included privacy concerns, misconceptions about CHW roles, and a preference for traditional medicine. Facilitators included strong CHW motivation, often driven by personal experiences with the conditions they managed. Community-level support, particularly from local leaders and sensitization events, enhanced intervention acceptance. At the health system level, training quality and role recognition of CHWs were critical, while barriers included excessive workload and insufficient infrastructure. Interpersonal relationships, especially gender dynamics and attitudes of facility-based workers towards CHWs, also influenced implementation outcomes. The quality of qualitative evidence varied, with many studies lacking clear objectives and data collection or analysis frameworks. Effective implementation of community-based interventions for chronic disease management in LMICs requires addressing both systemic and interpersonal barriers. Future interventions should emphasise structured community engagement, comprehensive training, and better integration with healthcare systems. Additionally, improving the methodological rigor of qualitative research is essential for gaining deeper insights into the complex factors that influence the success and sustainability of these interventions.

## Introduction

The rise in chronic diseases is a pressing health concern for health systems globally. Often subsumed under the umbrella term of non-communicable diseases (NCDs), chronic conditions account for 41 million deaths each year, of which 77% are in low- and middle-income countries (LMICs), and the vast majority occur prematurely [1]. Globally, health systems are not well equipped to systematically address chronic diseases, and although primary health care (PHC) is widely accepted as an effective way to prevent and control chronic conditions [2], PHC systems remain weak in many settings because of low investment and capacity [3]. In many low resource countries, lack of systematic screening and early detection, alongside low capacity at primary level and poor access to health care, lead to a situation whereby people with chronic conditions are often diagnosed late and require specialised care because of advanced disease. This places considerable burden on already stretched health services and their staff, with poor health outcomes among those affected [4].

Community-based interventions are initiatives that utilise local resources and the shifting of tasks traditionally performed by doctors to trained non-physician health workers (NPHWs) to address health needs directly where people live and work. Such interventions are increasingly viewed as an effective approach to address some of the capacity constraints faced in low resource settings. For example, a review of the evidence of effectiveness of NPHWs in managing chronic conditions in LMICs by Joshi et al. pointed to improved health outcomes, such as better control of hypertension, diabetes, and depression [5]. Several reviews specifically focused on the effectiveness of community health workers (CHW) in the delivery of chronic disease prevention and management, demonstrating that CHWs can effectively contribute to prevention such as smoking cessation and the control of blood pressure and diabetes [6–8]. A systematic review by Seidman and Atun found ‘substantial’ evidence that task-shifting can lead to cost savings and efficiency improvements in LMICs [9]. The evidence was mostly on task shifting activities related to tuberculosis and HIV/AIDS care, but their review also pointed to the potential for similar improvements to be achieved for chronic diseases at community level more broadly.

However, the sustainable implementation of community-based interventions can be challenging, with evidence reviews highlighting a range of barriers, such as restrictions on prescribing medications and the availability of medicines [5], as well as facilitators, including the quality of training of NPHWs, and familiarity of NPHWs with the local culture and language [7]. More recently, Heller et al., in their systematic review of reviews of health system barriers and facilitators to care provided by NPHWs for chronic diseases in LMICs identified six key strategies for effective NPHW integration: recruitment of qualified NPHWs, ongoing training and supervision, allowing NPHWs to prescribe medication and provide care, ensuring availability of medications and supplies, establishing reliable data systems, and ensuring a performance-based compensation to NPHWs [10].

While these reviews provide important insights into (some of) the implementation challenges of community-based interventions to manage chronic conditions in LMICs, much of the reviewed evidence focused on the effectiveness of task shifting in improving healthcare access and outcomes, with an emphasis on, largely, quantitative data. This often means that assessments of implementation barriers and facilitators lack depth in explaining how and why interventions succeed or fail in real world settings. Qualitative studies, by contrast, can illuminate the contextual, social, and relational dynamics that shape intervention uptake and sustainability. As emphasised by Varghese et al., understanding contextual factors and health system readiness is essential to ensure interventions involving NPHWs are effective and cost-effective [2]. Thus, there is a need for more nuanced understanding of implementation barriers and facilitators, using qualitative approaches to help inform future interventions that are appropriate and acceptable in a given local setting. The review of reviews by Heller et al. goes some way to do so, by including reviews of qualitative evidence [10]. However, only three of the 15 included reviews considered qualitative studies; overall, there was only a small number of qualitative studies, and these were primarily focused on mental health care. Furthermore, Heller et al. included reviews that were published up to July 2018, meaning that most recent primary studies included in those reviews likely date to 2017. Importantly, prior reviews also primarily examined health system-level challenges for integrating NPHWs, rather than interpersonal, community, and contextual factors that can shape implementation and these tend to be poorly captured by quantitative metrics.

This scoping review seeks to fill this important evidence gap by systematically mapping and thematically synthesising qualitative investigations of the barriers to and facilitators for implementing community-based interventions across a wide range of chronic conditions and settings in LMICs. Our focus is on providing rich, more granular insights into the multi-level barriers to and enablers of implementation across a range of intervention strategies, which includes task-shifting to non-physician workers as well as other types of community-based strategies.

## Methods

This scoping review was conducted and reported in accordance with the PRISMA-ScR (Preferred Reporting Items for Systematic Reviews and Meta-Analyses Extension for Scoping Reviews) guidelines to ensure transparency and methodological rigour throughout the review process (checklist shown in S1 Checklist).

### Search strategy

We searched six bibliographic databases from 2013 to October 2024: EMBASE (Ovid), Global health (Ovid), Medline (Ovid), Scopus, CINAHL Plus and Web of Science. The search strategies were drafted by an experienced researcher (SH) and further refined through team discussion. We used the following keywords: Implementation *AND* community-based interventions for diagnosis and management *AND* chronic conditions *AND* low-and-middle-income countries. The full search terms for MEDLINE are shown in S1 Text. We also conducted a manual search of the references cited in the included studies. The final search results were exported into EndNote, and duplicates were removed.

### Eligibility criteria

We considered original research studies that were published since 2013, had English language abstracts, and had a qualitative design or included a qualitative component examining facilitators for and barriers to implementing interventions. Studies that only reported quantitative measures of intervention effectiveness or implementation were excluded. Studies had to report on implemented or delivered community-based interventions. We defined ‘community-based’ as any place outside of healthcare facilities (primary, secondary, or tertiary), and which may include patients’ homes. The interventions had to be delivered by non-physician health workers and might include CHWs or nurses but excluded those delivered by medical health workers (e.g., doctors); we also excluded interventions that were self-administered or -directed (e.g., smartphone applications, text messaging). This review focused on interventions aimed at chronic disease screening, diagnosis, treatment, and control but excluded those aimed at behaviour change for control of risk factors (e.g., changes in physical activity, diet), or those aiming to solely increase linkage to health services. A chronic disease was defined as any persistent condition that requires ongoing management. As such, we also included interventions targeting people living with HIV. We considered low- and middle-income countries only, using the list of countries defined by the World Bank [11].

### Study selection

Articles were double screened by two independent researchers (SH and NK) using Rayyan software [12]. Disagreements regarding the eligibility of specific studies were resolved through discussion and consensus, and if necessary, a third researcher (EN) was consulted and made the final decision.

### Data charting

Data from eligible studies were extracted using a data abstraction tool developed by two researchers (SH and EN). One reviewer (SH) extracted data from all included studies, and a second reviewer (EN) verified a random subset of included studies for accuracy. The data extraction tool captured data on study characteristics; country of implementation of the intervention; setting (geographic area; health care facility type); study method (e.g., interviews, focus group discussions, etc.); sample characteristics (sample size, population sampling strategy); intervention characteristics and provider; reporting theories or frameworks; and facilitators and barriers to implementation of interventions of programmes.

### Analysis

We employed a two-stage approach to analyse and interpret the data that was reported in the results section of included papers. First, we conducted a thematic analysis to systematically categorise and synthesise the data extracted from included articles. Initially, data were coded in an inductive manner, to identify patterns and concepts. Codes were then grouped into potential themes, which were reviewed and refined through an iterative process to ensure that they accurately represented the data. In a second step, we used the Socio-Ecological Model (SEM) as a framework to interpret and structure our findings [13]. The SEM framework facilitated a comprehensive understanding of the data by examining factors at individual, community, and wider systemic and environmental levels. This strategy provided a means to articulate the multiple layers and interactions observed in the data, leading to a more nuanced understanding and reporting of the data.

We chose not to conduct a formal quality appraisal of the included studies, as the primary objective of this review was to synthesise themes and patterns across the literature. We prioritised the inclusion of a diverse range of studies to capture valuable insights and perspectives that might be otherwise be excluded if methodological rigor were strictly assessed.

## Results

### Study characteristics

**Fig 1:**
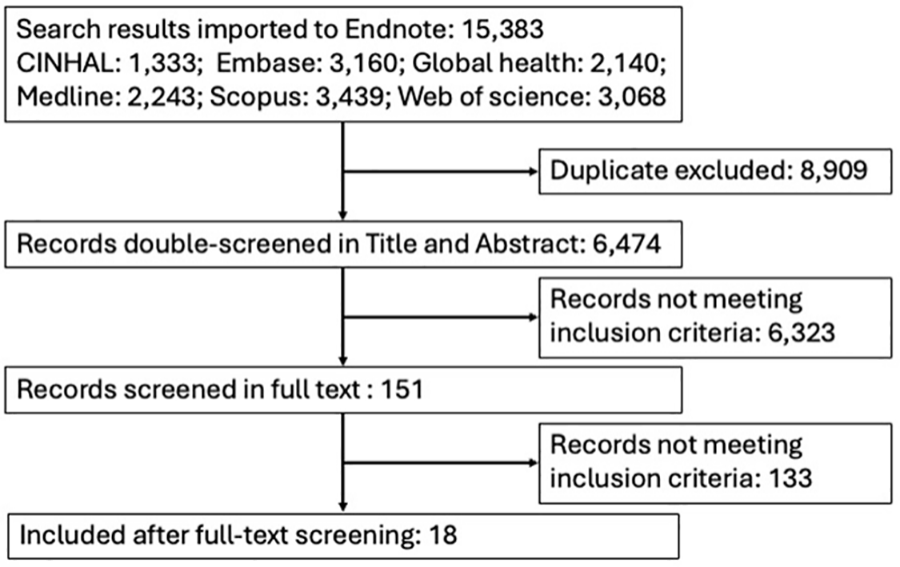
PRISMA flow diagram for study selection.

We identified a total of 15,383 records from database searches, of which 8,909 were duplicates. The remaining 6,474 were screened based upon title and abstract, of which 151 were retained for full text screening (Fig 1). Of these, 18 studies met our inclusion criteria and were reviewed. The manual search of references cited in included studies did not yield additional studies for inclusion.

Table 1 provides an overview of key characteristics of included studies. Of the included studies, nine were published before 2018, and 13 were published in 2018 or later. Studies covered 13 countries, most frequently South Africa (n = 4) [14–18], and India [16,19,20], Bangladesh [14,15,21], and Malawi (n = 3 studies each) [22–24], followed by Uganda (n = 3) [25–27], and Ethiopia (n = 2) [28,29]. Other countries include Nepal [30], Namibia [31], Sri Lanka [21], Pakistan [21], Brazil [16], Guatemala and Mexico [14–16]. Most studies reported on single-site interventions, with four studies examining multi-site interventions. Levitt et al. and Abrahams-Gessel et al. reported on different aspects of the same intervention implemented in Bangladesh, Guatemala, Mexico, and South Africa and we considered both studies to be pertinent to our objectives [14,15].

**Table 1:** Characteristics of included studies.

Authors	Country of intervention implementation	Principal Intervention	Method	Study sample and sample size	Sampling strategy/Recruitment
Jose, 2015	India	Non-communicable disease care	In-depth interviews and focus group discussions	In-depth interviews with 4 programme planners and managers, 19 programme implementers, 14 Accredited Social Health Activists, and 18 community members. 8 focus group discussions (number of participants not specified).	Not described
Abdel-All, 2019	India	Non-communicable disease care	A review of the current policies for NCD management and the Accredited Social Health Activist workforce. In-depth interviews and focus group discussions	In-depth interviews with 13 auxiliary nurse midwives, 7 medical officers, and 2 senior medical officers at the district level. 13 focus group discussions with 180 Accredited Social Health Activists, and 5 focus group discussions with 47 community members. *Total participants: 249*	Purposively selected 41 primary health centres for their geographic spread, with five Accredited Social Health Activists invited to participate from each. Sampling strategy not described for the remaining participants.
Ludwick, 2021	Ethiopia	Non-communicable disease care	In-depth interviews and focus group discussions	In-depth interviews with 18 programme administrators, 9 facility-based healthcare workers, and 13 urban health extension professionals. 2 focus group discussions with 12 urban health extension professionals. *Total participants: 52*	The study was carried out in two Ethiopian cities in two different states. Administrative lists obtained from the Regional Health Bureau and snowball sampling were used to recruit administrators. Recruited all family health team members in City 1. Recruited 9 family Health team members and administrators who were present at health centres vand offices during two days of visits in City 2.
Levitt, 2015	Bangladesh, Guatemala, Mexico, and South Africa	Cardiovascular disease risk screening and referral	Patient records from health centres in each site were reviewed for data on diagnoses made and treatment commenced, and qualitative data not further specified.	Study coordinators, community healthcare workers and the community members being screened for cardiovascular disease. Total number of participants for study data collection not specified.	Not described
Abrahams-Gessel, 2015	Bangladesh, Guatemala, Mexico, and South Africa	Cardiovascular disease risk screening, referral, and health education	Focus group discussion; Field notes from supervisors and study coordinators	Each site recruited at least 8–15 community healthcare workers to participate in the training. Total number of participants for study data collection not specified.	Not described
Ndejjo, 2020	Uganda	Cardiovascular disease risk screening, referral, and health education	Focus group discussion; Regular meeting reports during intervention implementation	5 focus group discussions of 20 community health workers. 41 reports.	Data were collected in five parishes that belonged to the first cluster of the stepped wedge (20 parishes in total received the intervention), at the end of the first intervention cycle of 6 months. During intervention implementation, community health workers had bi-weekly meetings with their community-based supervisor(s) who moderated the session and took notes on their experiences of implementing the intervention and wrote reports to capture these in addition to their own reflections and activities.
Safary, 2021	Malawi	Service integration of cardiovascular disease risk screening, referral, and health education with HIV infrastructure	Semi-structured interviews; Naturalistic observations	Interviews with 15 community health volunteers. 10 field observations.	Community health volunteers who were present at the community-based organisation site were interviewed and notes from field observations were conducted by the main researcher and community health nurse during the three-week data collection period.
Rawal, 2022	Nepal	Cardiovascular disease risk screening and health education	Focus group discussion	2 focus group discussions of 10 female community health volunteers.	Purposive selection of female community health volunteers was made through the discussion with the District Public Health Office. Four key criteria were used for recruitment: (i) working as a female community health volunteer in the study sites; (ii) having completed at least 8th grade of education or above; (iii) older than 18 years old; and (iv) voluntary willingness to participate in this study.
Teshome, 2022	Ethiopia	Hypertension screening	Semi-structured in-depth interviews	17 health extension workers, 7 immediate supervisors, 2 districts health office *Total participants: 26*	Purposive sampling was used to recruit one health extension worker from each of the candidate administrative units (kebele).
Jafar, 2023	Bangladesh, Pakistan, and Sri Lanka	Hypertension management (COBRA-BPS programme)	In-depth interviews	23 community health workers, 19 physicians and 45 hypertensive patients sampled from 15 intervention clusters (a cluster is 250–300 households defined by local administration according to community health workers catchment area). *Total participants: 87*	The sampling strategy was purposive and based on sex, age-group, and those who had already been recruited in the trial in randomly selected clusters.
Flor, 2020	Brazil, India, South Africa (and USA)	Hypertension and Diabetes management (HealthRise programme)	Key informant interviews and focus group discussions	37 participants in Brazil: 9 patients in focus group discussions. Interviews with 17 CHW, 8 facility or clinic managers and administrators, 3 policymakers. 19 participants in India: 5 patients in focus group discussion. Interviews with 3 community health workers, 3 facility-based or clinic-based providers, 4 facility or clinic managers and administrators. 15 participants in South Africa: 4 patients in focus group discussion. Interviews with 3 community health workers, 2 facility-based or clinic-based providers, and 1 facility or clinic managers and administrators. *Total participants: 71*	Qualitative data collection was conducted at a randomly selected subset of facilities in which quantitative data collection occurred. Sampling strategy not described.
Ingenhoff, 2023	Uganda	Home-based chronic obstructive pulmonary disease (COPD) screening and referral	In-depth interviews and focus group discussions	Interviews with 8 CHWs and 10 healthcare providers. 6 focus group discussions with 34 community members.	Purposive sampling of community members (varying in age, gender, COPD status, and referral adherence), CHWs working in COPD screening employed by the local non-governmental organization the African Community Center for Social Sustainability Uganda (ACCESS), and healthcare providers involved in screening, referral, or programme administration at ACCESS and Nakaseke District Hospital.
Kalumbi, 2014	Malawi	HIV counselling and testing	Exit interview questionnaire with questions in open-ended format	105 counsellors.	Consecutive sampling.
Ochom, 2018	Uganda	Service integration HIV counselling and testing with TB contact investigation	Focus Group Discussion	2 focus group discussions of 13 community healthcare workers.	This study was carried out in communities surrounding seven public primary care clinics providing TB services. At each clinic, 1–3 community health workers involved in TB-related activities were recruited.
Ngcobo, 2020	South Africa	HIV counselling and testing	Focus group discussions	4 focus group discussions of 36 community healthcare workers. 3 focus group discussions of 22 people living with HIV. *Total participants: 58*	Participants were purposively selected from 11 participating clinics.
Khumalo, 2022	South Africa	Community support for people living with HIV	In-depth interviews	11 outreach team leaders (supervisors of community health workers)	Purposive sampling of one outreach team leader, employed for one year or more, from each of the 11 districts.
Sande, 2020	Malawi	Community-based Anti-retroviral therapy programme	In-depth interviews, and observation	In-depth interviews with 4 community nurses and 18 patients. Observations took place on 3 days for a minimum of one hour (Patients-to-nurse interaction, time spent with the nurses and how patients interacted with each other were observed and recorded.) *Total participants: 22*	All four community nurses who provided the service in the patients’ communities
Katirayi, 2022	Namibia	Community-based Anti-retroviral therapy programme	In-depth interviews and focus group discussions	In-depth interviews with 40 patients, 8 health extension workers, and 11 policy makers and programme managers (3 at national level, 4 at regional level, 4 at district level). 4 focus group discussions with healthcare workers (number of participants not specified).	Patients either volunteered (convenience sampling) or were purposively selected if they were the first or last patients to receive services (to avoid losing their spot in the queue). Health extension workers were selected using convenience sampling. Sampling of policy makers not described.

Fourteen of the included studies were delivered by community health workers (CHWs), community health volunteers or health extension workers (henceforth referred to as CHWs) [14–18,21–23,25,26,28–30]; Sande et al. and Katirayi et al. described nurse-led interventions [24,31]. Jose et al. and Abdel-All et al. focused on Accredited Social Health Activist as a specific type of community health worker in India, who operate within India’s National Rural Health Mission, and typically receive performance-based financial incentives [19,20].

Among the included studies, 17 used semi-structured interviews, focus group discussions, or both. Additional data collection methods included documentary data from regular meetings between CHWs and supervisors [26], fieldnotes from supervisors and coordinators [15], non-participant observation [22,24], and an exit interview questionnaire with open-ended questions [23].

Studies varied in data collection scope and size. In six studies, data collection was limited to CHWs [15,22,23,25,26,30], two studies collected data from both CHWs and intervention participants [14,17], while the remaining ten studies involved a wider range of participants including CHWs, their supervisors, intervention recipients, facility-based health workers, programme managers, administrators, or policymakers [16,18–21,24,27–29,31]. Sample sizes varied significantly, ranging from 10 female community health volunteers in two focus group discussions [30], to 249 participants involving 180 Accredited Social Health Activists in 13 focus group discussions, 47 community members in 5 focus group discussions, and 22 in-depth interviews with auxiliary nurse midwives, medical officers, and senior medical officers at the district level [20].

### Intervention characteristics

Community-based interventions assessed in included studies varied in scope, target conditions, and delivery models (Table 2). For clarity, we present the interventions grouped by the primary condition or disease area they addressed.

**Table 2:** Characteristics of included interventions.

Authors	Country	Principal Intervention	Provider	Duration of intervention	Description of intervention
Jose, 2015	India	NCD screening integration into the National Rural Health Mission (NRHM)	ASHAs	Not specified	The NRHM was launched by the Government of India in 2008, aiming to provide accessible and affordable healthcare to rural and vulnerable populations. A pilot in Neyyattinkara Taluk aimed to integrate NCD screening into community outreach. ASHAs conducted burden assessment and Information Education and Communication (IEC) and follow-up, while Junior Public Health Nurses (JPHN) conducted screening for diabetes and hypertension at sub-centre level. The programme also ensured continuous medication supply through sub-centres.
Abdel-All, 2019	India	NCD control through the National Program for Cardiovascular Diseases, Diabetes, Cancer, and Stroke (NPCDCS).	ASHAs	Launched in 2010	The NPCDCS was launched in 2010 to target the rising burden of NCDs. ASHAs screened for chronic disease risk factors, conducted community surveys for family history, mobilised participation in screening days, promotoed healthy lifestyles, and followed up patients for medication adherence and continuity of care.
Ludwick, 2021	Ethiopia	NCD care through the Family health teams (FHTs) programme	An outreach team consisting of clinical professionals and CHWs	Launched in 2014–2015; with a subsequent rapid fade-out within a few months.	In 2014–2015, Ethiopia’s primary healthcare reform introduced Family Health Teams (FHTs) to deliver community-based care for low-income urban households . The team was led by clinical staff, accompanied by 5–6 CHWs, a social worker, and laboratory, pharmacy, and administrative personnel as required, and provided free health education, treatment, and referrals. CHWs were shifted to health centre-based supervision as part of the reform.
Levitt, 2015 and Abrahams-Gessel, 2015	Bangladesh, Guatemala, Mexico, and South Africa	CVD risk screening and referral	CHWs	4-6 weeks	CHWs from each country were trained to conduct CVD risk screening using non-laboratory tools, including blood pressure measurement; BMIcalculation; and obtain a 5-year CVD risk score for experiencing an adverse cardiovascular event. Screening took place in three settings: individual homes, at community events, or at self-help groups. Based on screening results, urgent or non-urgent referrals were made to local health facilities, with referral pathways adapted to each country. Health professionals independently validated risk scores to ensure appropriate referral decisions.
Ndejjo, 2020	Uganda	CVD risk screening and health education	CHWs	2 years	As part of the Scaling-up Packages of Interventions for Cardiovascular disease prevention in Europe and sub-Saharan Africa (SPICES) project, CHWs worked with existing community networks and structures to conduct home-based CVD screening using a non-laboratory tool, provide health education and promote lifestyle change through motivational interviewing and goal-setting. CHWs followed up with high-risk individuals after referral, but a formal referral pathway was not explicitly described .
Safary, 2021	Malawi	Service integration of CVD risk screening, referral, and health education into routine care and existing HIV testing and treatment infrastructure.	CHWs	Not specified	CHWs were given kits containing a bag, digital blood pressure machine, weight scale, height board, rubber stamp, and reporting books. CHWs conducted cardiovascular risk screening, BP measurement, referral of patients with elevated BP, following standard operating procedures and provided health education following standardised guidelines. They recorded findings with a rubber stamp template.
Rawal, 2022	Nepal	CVD risk screening and health education	Female community health volunteers (FCHVs)	2 months	FCHV conducted door-to-door visits to measure blood pressures and calculate a 10-year CVD risk using the 2019 WHO cardiovascular risk prediction charts.. They provided brief health education and a brochure containing information on CVDs risk and lifestyle modifications to reduce CVD risk.
Teshome, 2022	Ethiopia	Hypertension screening	Health Extension Workers (HEWs)	5 months	This was a diagnostic accuracy study. HEWs measured blood pressure during home vists. After a 5 min rest, trained health professionals, blinded to HEWs’ readings, repeated the measurements. Participants with high BP were referred to a nearby health care facility.
Jafar, 2023	Bangladesh, Pakistan, and Sri Lanka	COBRA-BPS (Control of Blood Pressure and Risk Attenuation-Bangladesh, Pakistan, Sri Lanka), a multicomponent hypertension management program	CHWs	2 years	The intervention consists of 5 components: CHWs provided education on hypertension management, lifestyle changes, and medication adherence during home vists. regular blood pressure monitoring by CHWs with referrals to healthcare facilities for those with high readings. Training of public and private healthcare providers in current hypertension management guidelines and best practices. Designated hypertension triage counters in government healthcare facilities = , managed by care coordinators to ensure patients receive timely and appropriate care. A financing model to support affordabile and sustainable access the hypertension services and medications.
Flor, 2020	Brazil, India, South Africa	HealthRise: pilot programmes aimed to improve screening, diagnosis, management, and control of hypertension and diabetes among underserved communities	CHWs	5 years (2014–2019)	The activities carried out in Brazil, India, and South Africa, activities were tailored to each local context, but shared common strategies that included CHWs training to use digital tools for NCD screening and educations. They conducted home visits,participated in community health events, and collaborated with local health networks and organisations to identify individuals at risk..
Ingenhoff, 2023	Uganda	Home-based chronic obstructive pulmonary disease (COPD) screening and referral	CHWs	6 months	CHWs conducted household COPD screening using peak flow meters from 3500 participants sampled from a census study. Individuals meeting referral criteria were referred to the District Hospital for spirometry testing.
Kalumbi, 2014	Malawi	Home-Based HIV Counselling and Testing	Trained cousellors	2 years	One volunteer living in the community, known as Resident Community Mobilizer,worked as a counselling aide supporting counsellors trained in home-based HIV counselling and testing to provide services systematically across the entire village.
Ochom, 2018	Uganda	Service integration of home-based HIV counselling and testing into household TB contact investigation	CHWs	1 year	CHWs delivered home-based HIV counselling and testing within a household-randomised controlled trial of home-based TB contact tracing. They provided group pre-testing HIV education, conducted testing, private post-test counsellingand referrals to HIV-positive contacts to their preferred clinic.
Ngcobo, 2020	South Africa	Home-based HIV counselling and testing	CHWs	CHWs carrying out HIV-related tasks in the community since the early 2000s. Study conducted between June and August 2019.	CHWs conducted HIV transmission prevention activities, including health education; identification and testing of those at risk; adherence support; and early identification of individuals with deteriorating health while on ART.
Khumalo, 2022	South Africa	Longitudinal patient support in communities for people living with HIV including education, referral to services, and ART adherence support.	CHWs	A policy framework to expand the role of CHWs introduced in 2018	CHWs supported educational campaigns and prevention programmes through distribution of condoms; promotion of HIV testing, safe sexual practices, and medical interventions like circumcision and STI treatment. They supported screening by identifying individuals at risk, referred patients for testing, assisting with accessing health services and referring to social and community services. They also supported adherence to treatment by providing counselling for antiretroviral therapy and enhancing treatment literacy.
Sande, 2020	Malawi	Community ART programme	Nurse	2 years	This nurse-led community ART programme (NCAP) was developed for patients who were adherent on ART. Eligible patients can access care in their communities instead of travelling to a fixed facility. Patients collaborate with the Community Health Services team to identify the place where they can receive care and collect ART during their appointment dates. Health promoters follow up with those who miss their appointments and provided feedback to the nurses.
Katirayi, 2022	Namibia	Community-based ART (C-BART) programme	Facility teams and HEWs	Intervention first implemented in 2006	Local community leaders provided land for C-BART sites and HIV-positive community members built and maintained the sites. A team from the facility comprised of registered nurses and a community counsellor visited a C-BART site bi-monthly to provide services (a data clerk, tuberculosis field officer, and/or pharmacist assistant would join if needed). Core services included ART prescriptions refills, HIV testing and counselling, clinical evaluations, blood specimen collections for viral load and CD4 analysis, and adherence counselling. Patients who missed appointments were followed up in by HEWs, who were community-based health assistants employed by the Ministry of Health and Social Services.

Thus, three studies described targeting multiple NCDs within two national programmes or broader health system reforms, where community-based services were a component of a wider strategy. Of these, two took place in India, and were delivered by Accredited Social Health Activists in India. The first sought to integrate screenings for cardiovascular diseases, diabetes, cancer, and stroke within the existing National Rural Health Mission [19], while the second was a National Program for Cardiovascular Diseases, Diabetes, Cancer, and Stroke that was launched specifically to target these conditions [20]. The third study by Ludwick et al. [29] examined the role of CHWs in chronic disease management and their integration with family health teams (FHTs) as part of Ethiopia’s national primary healthcare reform.

Five studies examined programmes for cardiovascular disease (CVD) risk screening, referrals, and health education implemented in different countries. All interventions involved CHWs who used non-laboratory CVD risk assessment tools and automated devices to measure blood pressure in the community. The multi-site intervention in Bangladesh, Guatemala, Mexico, and South Africa, included opportunistic screenings in people’s homes, at community events, or self-help groups and referral to either urgent or non-urgent further evaluation based on blood pressure measurements [14,15], while in Nepal and Uganda screenings were primarily done through home visits, and the intervention included health education [26,30]. In Malawi, the intervention included both referral and health education alongside screening, and it was integrated into an existing HIV testing and treatment systems [22].

Three studies specifically focused on screening for hypertension and/or diabetes. Teshome et al. [28] looked at diagnostic accuracy of blood pressure measurements taken by CHWs during home visits compared to those taken by health professionals in Ethiopia. Jafar et al. [21] evaluated a five-component hypertension management programme delivered through home visits by CHWs in Bangladesh, Pakistan and Sri Lanka, while Flor et al. [16] examined a programme to enhance the screening, diagnosis, management, and control of hypertension and diabetes in underserved communities in Brazil, India, and South Africa, delivered by CHWs in home visits. One study focused on household-based screening and referral for chronic obstructive pulmonary disease (COPD) delivered by CHWs in Uganda [27].

The remaining six studies assessed HIV interventions, including three studies of home-based HIV counselling and testing (HCT) conducted by CHWs [17,23,25], one of which was integrated into household tuberculosis contact investigations [25] Three further studies explored support strategies for people living with HIV, involving CHW-delivered follow-up, linkage to services, and adherence support [18], and nurse-led antiretroviral therapy refills in the community [24,31].

### Use of theories or conceptual frameworks

Only four of the included studies explicitly used theory or a conceptual framework to guide analysis. Jafar et al. [21] used the Theoretical Framework for Acceptability framework developed by Sekhon et al. [32] to assess the acceptability of the intervention being implemented and so inform its future scale up. The TFA considers seven components: affective attitude, burden, perceived effectiveness, ethicality, intervention coherence, opportunity costs, and self-efficacy. Ludwick et al. [29] and Ndejjo et al. [26] used the Consolidated Framework for Implementation Research (CFIR) to examine the different factors acting at organisational and system levels that may facilitate or hinder the implementation of the intervention in their respective contexts [33]. Ingenhoff et al. [27] applied the Implementation Outcomes Framework [34], which identifies eight key outcomes thought to be critical to evaluating implementation efforts in healthcare: acceptability, adoption, appropriateness, feasibility, fidelity, implementation cost, penetration, and sustainability.

Only one study used a formal reporting framework, the consolidated criteria for reporting qualitative research (COREQ), to report their study, focusing on three domains: research team and reflexivity, study design, and analysis and findings [28].

### Implementation facilitators and barriers

Using the SEM framework, we identified four levels of influences that shaped the implementation of interventions aimed at the management of chronic conditions as reported in reviewed studies. These were: (i) the individual level, i.e., participants who receive the intervention (service users) and health providers involved in its delivery (service providers); (ii) the community level as the setting in which those interventions are delivered; (iii) the health care system or policy level; and (iv) interpersonal factors between different actors. We report on each level in turn.

#### The individual level: Service users.

Ten studies reported on facilitators and/or barriers at the individual level for participants receiving the intervention [14–18,24,26,27,30,31]. Documented barriers to implementation included concerns about privacy, with fears of unintended disclosure of health conditions, misconceptions of disease and roles of CHWs in its management, and preferences for traditional medicine or practice.

**Privacy and confidentiality:** Privacy was identified as an important factor that could reduce the acceptability of an intervention for the service user by five studies [15,17,18,27,31].

In the context of HIV, the primary concern revolved around confidentiality, particularly the unintended disclosure of an individual’s HIV status through, for example, patients being seen to attend local, community-based antiretroviral therapy sites [31], or home visits by CHWs because CHWs were believed to only visit individuals who are seriously ill [17]. It was noted that patients may place greater trust in CHWs who are not from their immediate local area due to concerns about confidentiality and privacy and a concern that local CHWs might disclose their health status to others in the community. Additionally, there was a perception that CHWs from other areas would be more professional and less likely to judge, focusing solely on their job responsibilities [17,18]. Ngcobo et al. further highlighted that one significant barrier to CHWs providing HIV care in the community was patients giving incorrect home addresses as they preferred to visit clinics outside their local area rather than the nearest one because of fear of stigma. Stigma was also a concern in the context of screening for COPD, due to its perceived overlap with symptoms of infectious diseases, suggesting misconceptions about the nature and transmission of COPD. Studies also highlighted the importance of providing adequate physical space in community-based settings [31], as observed by Abrahams-Gessel et al. [15] in their study of a CVD intervention in Guatemala, and by Ingenhoff et al. [27] in their study of COPD screening in Uganda, where lack of private spaces in the local community centre made it difficult to maintain privacy during the screening process.

*“We had difficulties with that place … for example if a woman…needed to remove*

*her – güipil – (traditional clothing) … we didn’t have a private place....” – CHW in Guatemala, p.10* [15]

**Misconceptions about diseases and CHWs’ roles:** Six studies identified misconception about diseases and limited patient understanding of CHWs’ roles within health service delivery as important barriers to the effective implementation of community-based interventions [14,20,23,26–28]. For example Levitt et al. [14] reported that CHWs were often perceived only as basic health workers providing education or dispensing medication, with little appreciation for their broader roles. Furthermore, Ingenhoff et al. [27] reported that some community members showed distrust of the government, operating organisations, and CHWs. Ndejjo et al. [26] similarly found community mistrust toward CHWs motives, including assumptions about political or western interests, where CHWs were sometimes accused of being spies.

*“Some don’t want to tell us their age or about themselves. They see us as spies and they say, ‘why are you asking me all this, are you a spy? They usually don’t like so many questions. As you reply, you must handle them with politely. You can explain to them that I am a fellow community member, you cannot suspect that I can have bad intentions towards you. Then you go on to explain to them carefully that you are not a spy and that you are only interested in health issues. Then you sensitize them. Some of the community members are witnesses that what we have sensitized them about are good issues and they have worked for them.” FGD 2, CHW 2, p.10* [26]

Misunderstandings about chronic diseases such as hypertension, CVD, or COPD further complicated CHWs’ engagement with communities [20,26–28]. CHWs reported difficulties in convincing individuals to adopt preventive health behaviours or attend their follow-up appointments due to poor understanding of disease risks or lack of visible or debilitating symptoms, particularly for COPD [14,27]. In Uganda, COPD remains relatively unknown compared to other NCDs, with community beliefs about witchcraft contributing to misconceptions about the condition [27], while in the context of CVD, Ndejjo et al. reported that patients did not understand the relevance of measurements, such as waist and hip circumference, requiring time-consuming explanations [26].

*“The major barrier is poor community perception of the disease. That is, if you tell them you have HBP (high blood pressure) and don’t eat this, don’t drink this, don’t do this, and do exercise, they will not understand easily” Health extension worker supervisor, p.7,* [28]

Misconceptions extended beyond disease understanding to the perceived value of health interventions themselves, such as health education and counselling. For example, Kalumbi et al. [23], in their assessment of a home-based HIV counselling and testing intervention in Malawi, observed limited interest among people with HIV in pre- and post-test counselling, especially if testing results were negative, reducing the overall effectiveness of the intervention. Additional challenges included concerns over diagnostic procedures. In the case of COPD screening individuals were reported to have fears of their lungs “bursting” or disease spreading through testing equipment. Similarly, anxiety about test results and fear of pain were identified as barriers to HIV counselling and testing [23,27].

**Preference for traditional medicine:** Preference for traditional medicine in some communities, as described by Khumalo et al. in their study of patient support for people living with HIV in South Africa, led to resistance against HIV services provided by CHWs.

*“Communities don’t accept their [CHWs’] services because they believe in using traditional muthi [medicine]. They do not want to even hear what they want to tell them and that hinders the provision of HIV services that community care givers are bringing in their homes”*

*District 5, p.4,* [18]

However, Ingenhoff et al. [27] highlighted that preference for traditional medicine may not only be tied to cultural beliefs but could also reflect socioeconomic barriers. In their study, participants found COPD medication unaffordable relative to their daily wages, leading them to favour more affordable traditional or herbal remedies as alternatives.

#### The individual level: Service providers.

We identified two facilitators for providers delivering the intervention: intrinsic motivation and personal experiences of the disease they are treating.

**Motivation and commitment:** Eight studies highlighted the strong motivation and commitment of NPHWs, driven by various factors [15,19–21,26,29–31]. In Ethiopia, CHWs providing NCD care through the Family Health Teams felt respected and valued by their communities, which made them feel ‘happy’ and ‘lucky’ (City1-FGD1), p. 6 [29] Patient recognition and respect was also reported as a key motivator by CHWs in several other settings [15,19,21,26,30].

*“She is like our daughter; she visits me often, enquires about my drug intake and brings me when it is over. We can call her any time, because she lives nearby, and I wish if she could check my BP and then I could avoid going to hospital for checkup. She gives us Packet drink (ORS) for vomiting or loose stools.” A212-an elderly community member, p.21,* [19]

Non-financial incentives, such as t-shirts, training certificates, uniforms, and meals, can also act as important motivators for CHWs to deliver CVD risk screening and health education [26,30]. Ndejjo et al. [26] also noted that CHWs were motivated by the personal knowledge gained through training, along with invitations to attend project events, enabling interaction with other CHWs which were perceived to be inspiring.

*“Attending the project dissemination event uplifted and gave us some bit of change. It helped us analyse our performance because at first, we just worked for the sake but when we got to meet other CHWs and saw how they performed, it gave us more passion for what we do. I really felt so challenged that immediately we got back from that event, I started to work such that we can reach the level of the others.” FGD 2, CHW 1, p.12,* [26]

Rawal et al. [30] found that CHWs were motivated by their awareness of the rising prevalence of chronic conditions and their desire to help address these issues. Ndejjo et al. [26] and Abdel-All et al. [20] reported that CHWs were driven by a strong commitment to supporting their communities, motivated by positive behavioural changes, health outcomes, and favourable feedback from community members.

*“Cardiovascular diseases can be reduced. Those who are already suffering cannot be cured. But we can prevent those who have not suffered yet.” Female community health volunteer, p.9,* [30]

**Personal experiences:** Three studies highlighted the role of CHWs’ personal experiences of conditions that they managed, which positively impacted their service delivery and acceptance by the community. For example, CHWs with personal experience of HIV were perceived to better understand their patients [17], and where CHWs disclosed their HIV status this led to greater acceptance of their HIV testing and counselling services [18]. Similarly, Ndejjo et al. [26] observed that CHWs with CVD risk factors like hypertension or diabetes used their experiences to prompt change and generate interest in health interventions.

*“If they are also HIV positive, rather than (community health workers who are HIV-negative) just people who know nothing about what we have been through; someone going through the same experience won’t come to a household with an attitude.” PLWHIV_2, p.12,* [17]

#### The community level.

Five studies highlighted the importance of community-level actions, such as sensitisation through community-wide events and gaining support from local leaders, as key factors in facilitating health interventions [19,26–28,31].

**Community engagement and mobilisation:** In India, Jose et al. [19] observed that ward-level screening events for hypertension and diabetes increased acceptance of subsequent home visits by Accredited Social Health Activists for follow-up and medication support. The authors noted that participation of diverse community groups in these events boosted confidence in the health system and challenged the notion that government hospitals are only for the poor. Similarly, Ndejjo et al. [26] emphasised that media campaigns and community-based screening programmes raised awareness of CVD risk factors, reinforced CHW messages, and improved community compliance. Testimonials from community members encouraged further engagement, while negative experiences deterred involvement, fostering trust and engagement, while negative experiences deterred participation.

*“It is good that government thought about the poor diabetics in the rural areas who had difficulty in doing these tests and arranged the camps (ward-level community screening events) at our doorstep.” LA10-A local leader, p.20,* [19]

**Community leadership and influence:** Local leaders can also play a key role by facilitating access to resources and encouraging community participation. For example, leaders have helped secure land for community-based therapy sites or leveraged public events, like religious or community gatherings, to promote awareness and advocacy for health programmes [26,28,31].

*“The good thing is that we can raise the issue of hypertensive disease at the church on Sundays and holidays in collaboration with kebele [the smallest unit of local government, equivalent to a neighbourhood or ward] special organisations, namely the kebele administration and women’s organisation” (R04, HEW), p.6,* [28]

At the same time, as reported by Ingenhoff et al. [27], community leaders’ involvement can sometimes pose financial challenges for CHWs. Their study found that leaders routinely assisted CHWs by identifying the residences of study participants but often expected compensation for their efforts, creating additional financial burdens.

#### The health system and policy level.

At the health system and policy level, implementation facilitators included training and recognition of CHWs as essential healthcare providers, while barriers included inadequate remuneration and excessive workloads that are not aligned with CHWs’ responsibilities, a lack of standardised operating procedures and clarity in role definitions within healthcare teams, complicating the integration of CHWs.

**Training and supervision:** Training of service providers was provided in all community-based programmes except four [20,24,28,31]. In two studies, where training was not done, its absence was reported as a major barrier to implementation [20,28]. Ingerhoff et al. additionally noted ongoing capacity building and supervision for CHWs as an important factor for intervention sustainability [27].

Nine studies provided information on training content, of which five emphasised interactive and practical components with supportive supervision as key facilitators [14,15,23,25,26], and five identified inadequate duration of training as a main barrier [15,19,22,29,30].

For example, the multisite CVD risk screening and referral intervention reported by Levitt et al. [14] and Abrahams-Gessel et al. [15] included site-specific training of CHWs over one week (Box 1). Training duration and modality were adapted in response to trainer and trainee feedback (*‘It was a lot of information for four days and in such a traditional format’* (CHW, Mexico), p.8, [15] and this change was seen to have enhanced learning and, ultimately, trainee performance.

Box 1 **CHW training components for CVD risk screening and referral in Bangladesh, Guatemala, Mexico and South Africa [15]**

The CHW training was one weeklong, and combined practical and didactic components, which involved:

- Practical training in CVD risk factor assessment (blood pressure measurement, body mass index, CVD risk scoring).
- Ethical conduct, including confidentiality and informed consent.
- A training manual in local languages.
- Workshops, including lectures and interactive lessons delivered by health professionals.
- Continuous supervision and weekly performance assessments provided by study coordinators.

Training was adapted in response to participant (trainer, trainee) feedback using focus group discussions capturing CHWs’ experiences and pre-training, post-training, and post-fieldwork knowledge tests. Adaptations included extending training duration from an initial one week to two weeks, and a move to interactive exercises, role-playing, and case studies, which were tested in the South Africa site showing improved participant performance. Participants in the Mexico site indicated a preference for verbal or practical assessments by supervisors over written knowledge tests.

Likewise, a community-based CVD risk screening and health education intervention in Uganda reported by Ndejjo et al. [26] involved a multi-component training programme of five half-day training sessions and a two-day field orientation, which included frequent supportive supervision, regular feedback, simplified materials, and reinforcement through fieldwork. Regular meetings focused on goal setting, progress reporting, barrier identification, interactive sessions with supervisors, and refresher training, with CHWs support one another and actively sharing experiences with each other to foster learning and collaboration. Together, these activities were seen to be facilitating intervention implementation, and to empower CHWs with the knowledge, skills and confidence needed to engage.

*“The bi-weekly feedback meetings give us a platform to ask about things we do not know. Even after training, someone can keep at the same level of knowledge. However, during the meetings we get enough time to discuss and learn more rather than having phone calls when things might not be explained in detail.” (FGD 1, CHW 2), p. 11,* [26]

Similar observations were reported for community-based HIV testing and counselling interventions in Uganda [25] and Malawi [23], where training that involved interactive elements and field experience, allowing for discussion of sensitive issues and cases, alongside continuous supportive supervision, to be key to enhance CHWs’ confidence and knowledge and, thus, effective programme implementation.

*“it (training) helped me to be friendly and tolerant with people in the community so as to make the work easier.” Exit interview questionnaire of community counsellor, p.3,* [23]

*“You must understand that sometimes the people in the communities test them [CHWs] to see how much they know. So, I always avail myself to teach community care givers about HIV concepts like CD4 count, viral load and PrEP so that when they approach households, they do so with confidence. In return communities respect them when they can answer their questions” Outreach team leader in District 3, p.5,* [18]

Not surprisingly, short training duration, often involving only a few days, and inadequate training manuals were identified as key barriers to implementation in many settings, particularly where CHWs were expected to learn comparatively complex procedures such as measuring blood pressure and blood sugar for CVD screening and data recording [15,19,22,23,29,30].

*“We are a grass root level health volunteers and cannot claim to be able to remember and achieve everything after one training session. We have been helping women and children in the past through other projects. We are confident that we can also serve if we get a chance to learn more about hypertension” Community health volunteer, p.10,* [22]

**Workload and renumeration:** Excessive workload and inadequate compensation for CHWs were reported as barriers to implementation in ten studies [15,19–21,23,24,26–28,31]. CHWs often face expanding responsibilities, including chronic disease screening and follow-up, without proportional financial support or logistical facilitation.

Abrahams-Gessel et al. [15] noted that supervisors often underestimated the time required for CHWs to perform chronic disease management tasks, exacerbating their workload. Ingenhoff et al. [27] found that CHWs in rural areas struggled to locate participants, which prolonged already lengthy COPD screening procedures and further increased their workload. Ndejjo et al. [26] and Jafar et al. [21] reported that the high workload associated with chronic disease management detracted from CHWs’ ability to perform routine tasks. CHWs also described the additional demands as disruptive to their personal lives.

*“We get pressurised to finish more tasks and put aside our family needs... We end up getting blamed by our families, or even losing the second job we have”. Accredited social health activist, p.5,* [20]

The lack of adequate financial support for CHWs, which undermined motivation, was highlighted in several studies. For example, delays in payment due to logistical issues [19], lack of compensation for training sessions [23], or withdrawal of certain allowances, such as lunch packs and bush allowances for work in remote settings [31]. Ingenhoff et al. [27] highlighted that rising inflation further amplified financial strain. In India, Accredited Social Health Activists (ASHAs) were assigned additional tasks for chronic disease screening and follow-up resulting in a higher workload that was disproportionate to the financial compensation received [20].

**Standard operating procedures (SOPs):** Two studies highlighted the importance of establishing standard operating procedures (SOPs) for performing intervention tasks and community outreach [22,28]. The absence of hypertension management guidelines led to significant challenges such as inconsistent patient identification, with CHWs asking different questions or phrasing the same questions in varied ways, leading to different interpretations. Additionally, height boards and weight scales were often placed on uneven surfaces, resulting in inconsistent measurements. Despite having a BMI chart, CHWs omitted BMI calculation. Patients were informed of their blood pressure readings but were not provided explanations. Some CHWs provided brief lifestyle counselling, while others gave advice beyond their mandate on food and nutrition. Additionally, the lack of a formal referral and follow-up procedure hindered the tracking of patients between health facilities and the community [22,28]. Safary et al. [22] additionally noted the absence of SOPs for community sensitisation and outreach, causing CHWs to rely on informal networks like social groups and community leaders. While these networks can be beneficial, they may lead to variations in service reach and quality, undermining the effectiveness of chronic disease management interventions.

**Recognition and role definition for CHWs:** Two studies reported that recognising CHWs as integral members of the healthcare system was an important facilitator for intervention implementation and community acceptance [17,30]. Having a uniform was reported to be important for CHWs as it enhanced their credibility and facilitated household access by demonstrating their healthcare affiliation [17,30]. Ngcobo et al. [17] further noted that people living with HIV were more likely to trust CHWs if they had previously encountered them in a formal healthcare setting, highlighting the benefits of CHWs being recognised as part of the wider healthcare system.

*“Before they were not opening for us, but since we started doing health talks at the clinic and introducing the role of WBOTs, they now welcome us to their households.” Ward-based outreach team member 4, p.11,* [17]

Three further studies pointed to the importance of defining clear roles for CHWs within healthcare teams, and of establishing clear protocols for managing referral, communication and collaboration between CHWs and healthcare professionals, particularly for chronic care [20,22,29].

**Operational challenges and system integration:** Effective healthcare delivery relies on well-coordinated operations, and eight studies reported poor coordination and systemic inefficiencies, including poorly organised teams, inadequate time management leading to insufficient household visits, inadequate patient preparation by facility staff for NPHW services, and reluctance of facilities to accept NPHW referrals [16–18,24,27–29,31].

Reporting on a COPD screening programme, Ingenhoff et al. [27] found fragmented and time intensive referral process, with community members reported to be confused about referral processes and having difficulty finding the healthcare providers they were meant to consult, leading to lack of future follow up. CHWs were not informed about whether referred community members reached the referral site to better understand discrepancies between referrals and no-shows. Similarly, Ludwick et al. [29] highlighted that poor team coordination and resource mobilisation within family health teams providing community-based chronic disease care in Ethiopia were partly due to facility-based health workers perceiving community outreach services as supplementary rather than integral to their responsibilities. Additionally, A lack of local ownership and political commitment further undermined the program’s effectiveness. The programme was seen as a federally imposed initiative with unclear resourcing and governance structures, which failed to engage and motivate local leadership, and led to insufficient local ownership and buy-in.

*“There was no one to take responsibility for handling the FHT implementation. Only [NGO] was working with us”. (City1, Facility-based healthcare worker 1), p.1466,* [29]

Several studies pointed to the role of existing infrastructure and programmes in supporting intervention implementation [16,17,24,28]. For example, Flor et al. [16] in their study of a community-based intervention for the detection and management of diabetes and hypertension in four countries, noted that existing structures such as CHW-led service provision, community engagement and information systems had facilitated implementation in the Brazil study site, while these structures had to be developed from the ground in study sites in India and South Africa. Teshome et al. [28] highlighted the potential of leveraging ongoing campaigns, such as community vaccination and health insurance enrolment, as a means to enhance hypertension screening outreach.

*“… we have a campaign that will run alongside, and we will be able to reach them during the campaign. Therefore, it is a good opportunity to work together with the campaign” (R06-Health extension worker), p.6,* [28]

**Infrastructure and resource limitations:** Seven studies reported significant infrastructure and resource limitations as barriers to implementing interventions, including shortages of equipment and medical supplies, inadequate transportation, and insufficient site infrastructure [15,18,19,21,24,26–28,31].

Issues with equipment included insufficient diagnostic tools such as glucometers and blood pressure devices, broken tape measures, as well as the lack of calculators to assess risk scores [26,27]. Shortages extended to the workforce, with insufficient numbers of trained CHWs unable to cover large catchment areas, further limiting intervention reach [18,27]. In Ethiopia, CHWs reported buying medicines out of pocket for patients who were “powerless to buy drugs and are crying out for help” (City 2, *Facility-based healthcare worker* 1), p.1465, [29]

Unreliable or insufficient transportation frequently delayed NPHWs in reaching their assigned locations [18,24,31]. Ingenhoff et al. [27] reported that transport allowances were insufficient, especially in the context of increasing fuel prices in Uganda, forcing some CHWs to cover expenses out of pocket, with female CHWs being disproportionately impacted as they typically do not own or drive motorcycles in the study area and often had to pay additional costs for drivers. They also noted that although some CHWs were employed far from their place of residence, they received the same transport allowance as those assigned closer to their home village. Katirayi et al. [31] emphasised the need for vehicles equipped for specialised tasks, such as storing samples or carrying water tanks to maintain hygiene standards, during ART delivery. Additionally, better infrastructure was needed to support both privacy and functionality at community outreach sites, including protected waiting areas and secure spaces for physical examinations [15,31].

#### Interpersonal relationships.

Interpersonal relationships between CHWs and patients, as well as between CHWs and facility-based health workers, were found to significantly shape intervention implementation. Key aspects included the role of gender, boundaries between patients and CHWs and attitudes of facility-based health workers.

**The role of gender:** Three studies highlighted gender dynamics as playing a significant role in service implementation. Ndejjo et al. [26] and Abrahams-Gessel et al. [15] noted the need for CHWs to have a good understanding of gender norms, such as providing privacy when removing clothing for blood pressure measurements and addressing discomfort when taking waist and hip measurements of the opposite gender.

*“When I disapproved of her repeatedly working mostly on women, the CHW said she was uncomfortable fastening a tape measure around men as it would look like they are being touched to evoke sexual feelings. She also noted that this form of act was disapproved of in the Kiganda [local] culture. I advised her to explain to them [men] why she needs to take their waist and hip measures and where necessary solicit the support of another family member to support taking the measurements.” (CHW Supervisor report), pg.10,* [26]

Two studies highlighted challenges with CHWs reaching young people and men. Men were often absent during daytime visits, especially in mobile fishing communities, were less likely to seek clinic services, and viewed health interventions as more relevant to women, deferring CHWs to their wives, while young people saw these interventions as more pertinent to older individuals [17,26]. This points to the need for interventions to be mindful of gender and age when recruiting CHWs to enhance intervention efficacy in certain communities.

*“The CHW noted that capturing men was still a challenge, citing that most men always tell her that such programmes are for women and in any case, interviews and other data can equally be best provided by their wives. She stressed that you can find both the wife and her husband, and the husband tells you directly: ‘that is for women, I even don’t have time; you ask, screen and discuss anything with my wife’. On the other hand, some individuals claim that they don’t have any signs and symptoms of being unwell especially the youth.” (CHW Supervisor report), p.11,* [26]

*“You see door to door [ward-based outreach team] is good because some of us are afraid to come to the clinic as most man [men], they don’t like coming to the clinic.” (PLWHIV_2), p.5,* [17]

**Maintaining professional boundaries and trust between patients and CHWs:** Maintaining professional boundaries can be challenging for NPHWs as they build closer relationships with the community. Sande et al. [24] found that increased time with patients in a community nurse-delivered ART programme fostered stronger interpersonal relationships and helped build trust, but made it harder to maintain professional boundaries between themselves as a provider of services and the patient as service users. For instance, patients often arrived late for appointments or expected highly flexible service delivery, which disrupted the nurses’ ability to manage their tasks effectively

“*People get used to the fact that we will go to the community [and] come whenever they wish […] we are in one-to-one contact, [patients] tend not to be serious because there is interpersonal relationship, unlike at the clinic they meet different [health care providers]. They would not approach them the way they do in the community.” (Nurse 4), p.5, [*24*]*

Ingenhoff et al. [27] reported that personal conflicts between participants and CHWs could negatively impact community acceptance of screening services. Additionally, the perceived “bad social behaviours” of CHWs was seen to erode trust among community members, further hindering the effectiveness of CHW-led initiatives.

**Attitude of facility-based health workers toward CHWs:** Five studies identified implementation challenges due to concerns of facility-based healthcare workers about CHWs’ ability and skills to ensure competent and safe delivery. Healthcare staff acknowledged the essential role of CHWs in bridging linguistic, cultural, and geographic barriers, and providing extended counselling beyond the scope of typical clinical appointments, as reported by Flor et al. [16] in their chronic disease management study in Brazil, India, and South Africa. However, staff often questioned CHWs’ competency in delivering chronic disease services. Abdel-All et al. [20] noted that in India, Accredited Social Health Activists delivering chronic disease screening and follow-up faced harassment and disrespect from the primary healthcare team due to doubts about their skills *“when we take a patient to the hospital, they would tell us “you are an ASHA stay outside” (Accredited Social Health Activist), p.4,* [20] Levitt et al. [14] found that in Bangladesh, Guatemala, Mexico, and South Africa, CHWs delivering CVD risk screening and referral were seen as a threat by existing health professionals, likely referring to the perceived professional threat that CHWs may pose to their roles, authority, or status. Furthermore, CHWs’ referrals of high risk patients to health clinics for further assessment were not trusted. Similarly, in South Africa, healthcare professionals were reluctant to accept HIV patient referrals from CHWs.

*“Two days back we referred a client, but they come back to us saying that the staff refused to take the referral.” (Ward-based outreach team member_2, Ngcobo*

## Discussion

This scoping review identified key barriers and facilitators affecting the implementation of community-based interventions for chronic disease management in LMICs. By employing the SEM framework, we identified and explored challenges at the individual, community, healthcare system, and interpersonal levels. Our findings align with existing literature, such as those by Heller et al. (10) and Perry et al. (32), which discuss challenges like inadequate financing, including lack of supplies and inadequate compensation of NPHWs; suboptimal training and supervision; and the lack of clear roles and integration within healthcare systems. However, our review uniquely contributes further depth to existing literature by focusing on qualitative studies that addresses the implementation challenges of interventions for chronic conditions and uncovering specific, often overlooked, but crucial factors that influence the sustainable implementation of these interventions. These nuanced insights emphasise the importance of addressing even the more routine aspects of implementation to ensure long-term success.

This review found that while financial incentives were important in motivating NPHWs to engage in the delivery of community-based interventions, the trust and respect that they received from community members was an equally important motivator, as was personal experience of the diseases they managed and a desire to reduce the disease burden in their communities. This awareness and understanding enhanced NPHWs’ ability to understand patients’ experiences and helped build trust and deliver acceptable services, thereby facilitating intervention implementation. At the same time, being a member of the community can hinder effective implementation where familiarity blurs professional boundaries, leading patients to expect flexible service delivery from NPHWs, or, conversely, people not wishing to engage with local NPHWs on grounds of confidentiality or personal conflict. These considerations suggest that training for providers of community-based services should not only equip them with the skills to deliver the actual services, but also broaden their knowledge on disease trends and burdens and equip them with techniques to enhance communication and strike a balance in maintaining their credibility and professionalism while working closely within the communities they serve. This is consistent with other studies that also pointed to the importance of training that goes beyond clinical skills to include emotional support, cultural competence, practical assistance, and navigating complex relationships [35,36].

Our findings underscore the critical role of patient perceptions and community awareness in the effective implementation of community-based health interventions. Misconceptions about disease risks, perceived procedural harms, and the roles of NPHWs in disease management were significant barriers. Patients unfamiliar with the tasks performed by NPHWs may distrust the services they receive. Community events and media communication campaigns that promoted the implemented interventions could sensitise the public, for example, by preparing community members for household visits by service providers or reinforcing the health messages delivered by them. Additionally, engaging local leaders and organisations could help overcome some of these challenges. We found that engaging local leaders improved the acceptance and sustainability of interventions by mobilising resources, shaping public perceptions, and advocating for community-wide participation and ownership in health activities. Furthermore, while we applied the SEM framework to distinguish between community-level and health system-level influences, we acknowledge that in many settings, these boundaries are not always distinct. Community leaders often influence the operations of local health facilities, whether through advocacy, informal oversight, or facilitating resource mobilisation, and thus play a dual role across levels. This interdependence underscores the importance of cross-level collaboration and contextual sensitivity when designing and implementing community-based interventions. However, community leaders may also expect compensation for their time, highlighting the tension between leveraging community leadership and ensuring its sustainability. This observation underscores the need for programme designers to consider not only the logistic and strategic benefits of involving community leaders but also the economic realities and opportunity costs they face. Failure to address such expectations could lead to disengagement, as leaders might perceive their contributions as undervalued. These findings suggest that a multifaceted approach that incorporates structured community engagement, including of local leaders, and sensitisation activities, along with guidance on their effective implementation, should be a crucial element of community-based interventions. These activities should enhance public education on disease risks and prevention and emphasise the importance of NPHWs in health promotion, clarifying their roles and the scope of their work. Furthermore, there is robust evidence indicating that community engagement positively impacts various health outcomes and conditions [37,38]. However, in this review, we identified only five studies that referenced community engagement. Moreover, in these studies, community engagement was mentioned briefly and informally, without being reported or described as a distinct and explicit component of the intervention. This highlights a significant gap in the literature and underscores the need for more detailed and systematic reporting on community engagement strategies in future research.

Our findings also suggest that gender dynamics could significantly influence the implementation and success of community-based health interventions. Studies highlighted the need for CHWs to navigate cultural sensitivities, such as respecting privacy during health assessments and addressing discomfort with cross-gender interactions. These challenges point to the importance of gender-sensitive training and tailored communication strategies. Men and young people remain harder to reach, with men often perceiving health interventions as targeted at women and youth, suggesting the need for targeted recruitment and messaging to engage these groups. Additionally, transportation challenges could disproportionately affect female CHWs, who often lack access to transport vehicles and could incur higher costs. Addressing these barriers through gender-sensitive policies, equitable transport solutions, and inclusive recruitment can enhance the reach and effectiveness of community health programmes.

Our findings align with the broader literature emphasising the importance of training for interventions involving NPHWs for effective implementation [5]. However, to be effective and support sustainable implementation, our review indicates that training needs to be multi-faceted, and include practical elements and fieldwork, ongoing and supportive supervision, and mutual learning opportunities [15]. Indeed, Hamilton and Friesen suggest that methods which assess learning using standardised tests often overlook the broader, more practical aspects of the learning experience that are important to learners and fail to capture the full scope of what learners value and need [39]. Our findings also emphasise the value of regular meetings and supervision between supervisors and NPHWs, which facilitated discussions of sensitive issues, experience sharing, and ongoing support. This aligns with a systematic scoping review by O’Donovan et al., which reported positive outcomes of ongoing training of NPHWs in LMIC [40], although most the reviewed studies focussed on training for maternal and child health or infectious diseases, and there remains a lack of understanding of how to best implement ongoing training for the management of chronic conditions. Our findings further suggest that there is a need to systematically invest in NPHWs’ professional development, not only to enhance their performance, but also their motivation to perform their roles. Finally, training programmes should be appropriately timed to cover training materials effectively. For example, studies have identified challenges in understanding and explaining CVD risk scoring, indicating that complex material requires more practice and time to master [15,30].

A significant barrier to intervention implementation was a high workload of NPHWs, often due to supervisors underestimating the time required for chronic disease management tasks. This was exacerbated by unclear roles and responsibilities, where NPHWs were expected to take on additional tasks beyond their routine activities without clear guidelines or adequate support. Such assumptions about NPHWs capacity, often based on the notion that ‘simple tasks’ can be ‘shifted’ to them across a wide range of health areas with minimal training to relieve clinical providers, can create unrealistic expectations and lead to burnout. Large catchment areas and limited transportation further increased burden, particularly for female NPHWs who may face mobility constraints. A recent systematic review and meta-analysis showed that high workloads can increase burnout in health workers, defined as emotional exhaustion that leads to negative attitudes and behaviours [41]. Incorporating practical elements and fieldwork into training programmes can provide NPHWs with hands-on experience, helping them and their supervisors better understand the actual time needed for various tasks and develop more accurate time estimates and realistic job scopes for chronic disease management and other responsibilities. Ongoing supportive supervision could also reduc the risk of burnout [41]. Facility-based healthcare workers are integral in providing this supportive supervision, as well as in preparing patients for community services. Therefore, this cadre of healthcare workers should be supported in. Training should therefore extend to facility-based healthcare workers, to ensure they recognise the value of community outreach activities and the critical role of NPHWs, and to equip them to effectively supervise and support NPHWs, thereby fostering an environment of trust, respect, collaboration, and cross-learning. This is particularly important in resource-poor settings where supervision is not always formally recognised or compensated.. Increasing appreciation and understanding of community services and providers among all health system actos is essential to drive their intrinsic motivation to support these interventions.

### Strengths and weaknesses

We used a broad search strategy to identify studies of the implementation of community-based interventions for the management of chronic diseases in LMIC. This inclusive search strategy ensured that our review encompassed a diverse range of community-based interventions. However, defining precise search terms for community-based interventions proved difficult, potentially affecting the comprehensiveness of our literature search. Community-based interventions encompass a broad and diverse range of activities, settings, and target populations, making it challenging to capture all relevant studies using specific keywords. This difficulty may have resulted in the exclusion of pertinent studies that did not explicitly use the terms we searched for, thereby limiting the scope of our review.

By focusing on qualitative studies, our review provides in-depth insights into the implementation challenges and facilitators of community-based health interventions. Qualitative data allowed us to capture nuanced experiences and perspectives from various stakeholders, offering a detailed understanding of the complexities involved in intervention delivery. Although we did not conduct a formal quality appraisal of included studies, we recognise this as a limitation, and acknowledge that the quality of included evidence varied considerably. For example, although reporting on implementation challenges of interventions, included studies often did not clearly define their objectives as addressing implementation challenges or facilitators, requiring us to infer these aspects from the data. Furthermore, even when exploring implementation challenges and facilitators was defined as the objective of the study, many did not use conceptual or theoretical frameworks to inform their data collection or analysis. Frameworks provide an important means in the evaluations of complex interventions for systematically collecting and interpreting findings [42]. This lack of clear purpose and structure in reporting may have resulted in fragmented insights that are harder to integrate into a comprehensive and cohesive understanding of the implementation processes. Additionally, many studies lacked clarity in their methodological reporting, requiring us to infer specifics about data collection and analysis. These methodological limitations affect the synthesis of findings, as it introduces uncertainty and potential bias. Nevertheless, excluding these studies based on methodological criteria could have omitted valuable context-specific perspectives. This highlights the need for future qualitative research on community-based interventions to be more methodologically rigorous and transparently reported.

## Conclusions

Effective implementation of community-based interventions for chronic disease management in LMICs requires attention to multi-level barriers and enablers. At the individual level, interventions should incorporate clear, culturally sensitive health education to address misconceptions about disease risk and treatment and improve the acceptability of services delivered by NPHWs. At the interpersonal level, training should not only equip NPHWs with clinical knowledge, but also prepare them to navigate relational dynamics, such as stigma, gender norms, and maintaining professional boundaries. At the community level, future research should focus on developing and integrating structured community engagement strategies, including involving community leaders into interventions, and targeting harder-to-engage demographics such as men and younger populations, as these groups tend to be less engaged in preventive health services and may experience delayed diagnosis and treatment. This includes clear guidance on how to design, implement, and evaluate these strategies. At the health system and policy level, our findings underscore the need for multi-faceted training programmes that emphasise practical, hands-on experience, and extend beyond clinical skills to include cultural competence and techniques for managing complex relationships. Additionally, the review highlights the importance of ongoing supportive supervision and further research should explore its impact on reducing NPHW burnout. Research should explore best practices for integrating facility-based healthcare workers into community interventions, particularly through extending training to enhance their capacity to supervise NPHWs effectively and foster an environment of collaboration and cross-learning. Our findings also underscore the need for creating supportive work environments by clarifying the roles and responsibilities of NPHWs to improve workload, and motivating CHWs through non-monetary incentives, such as professional growth opportunities and recognition of their contributions. Further research should assess how these factors influence job performance, retention, and satisfaction. To improve the methodological rigor of studies, researchers should use established conceptual or theoretical frameworks to guide data collection and analysis, ensuring a systematic and comprehensive examination of implementation processes and outcomes. Clear definition of study objectives, detailed reporting of methods, adequate sample sizes, and triangulation of data sources are essential for enhancing the credibility and validity of findings. Additionally, there is a need for much more robust implementation research that employs qualitative approaches to capture the complexities and nuances of real-world settings, providing deeper insights into the factors influencing intervention success and sustainability.

## Supporting information

S1 Checklist Preferred Reporting Items for Systematic reviews and Meta-Analyses extension for Scoping Reviews (PRISMA-ScR). (DOCX)

S1 Text MEDLINE search terms. (DOCX)
