# Supplementary material for: Implementing community-based interventions for the management of chronic conditions in low- and middle-income countries: A scoping review of qualitative evidence
Source: PLOS Glob Public Health. 2025 Jul 8;5(7):e0004860. doi: 10.1371/journal.pgph.0004860 (PMC12237052; doi:10.1371/journal.pgph.0004860)
Supplement: S1 Text — (DOCX) [file pgph.0004860.s002.docx]

S1 Text. MEDLINE search

1. (implement* or deliver* or evaluat* or quality or monitor* or adopt* or barrier* or limit* or facilitat* or feasibil* or fidelity or adapt* or compatib* or suitab* or appropriate* or sustainab* or scale* or accept* or satisfact* or access* or lesson* or generalis* or utili* or use* or reach* or applica* or experienc* or motivat* or perform*).mp. [mp=title, book title, abstract, original title, name of substance word, subject heading word, floating sub-heading word, keyword heading word, organism supplementary concept word, protocol supplementary concept word, rare disease supplementary concept word, unique identifier, synonyms, population supplementary concept word, anatomy supplementary concept word]

2. (communit* adj3 (health or healthcare or "health care" or "health-care" or professional* or physician* or clinician* or worker* or provider* or volunteer* or based or led or intervention* or program* or approach* or level* or service* or manag* or treat* or therap* or investigat* or detect* or test* or diagnos* or screen* or adher* or support* or prevent* or engag* or participat* or outreach*)).mp. [mp=title, book title, abstract, original title, name of substance word, subject heading word, floating sub-heading word, keyword heading word, organism supplementary concept word, protocol supplementary concept word, rare disease supplementary concept word, unique identifier, synonyms, population supplementary concept word, anatomy supplementary concept word]

3. Developing Countries/

4. ((developing or "less developed" or underdeveloped or "under developed" or "under-developed" or underserved or "under served" or "under-served" or deprived or poor) adj (econom* or setting* or countr* or population* or context* or area*)).ti,ab.

5. ((low* or middle) adj3 income).ti,ab.

6. transitional countr*.ti,ab.

7. global south.ti,ab.

8. "africa south of the sahara"/

9. africa, central/

10. africa, eastern/

11. africa, southern/

12. africa, western/

13. ("Africa South of the Sahara" or sub-Saharan Africa or subSaharan Africa).ti,ab.

14. Central Africa.ti,ab.

15. Eastern Africa.ti,ab.

16. Southern Africa.ti,ab.

17. Western Africa.ti,ab.

18. "Democratic People's Republic of Korea"/

19. (North Korea or (Democratic People* Republic adj2 Korea)).ti,ab.

20. Cambodia/

21. Cambodia.ti,ab.

22. Indonesia/

23. (Indonesia or Dutch East Indies).ti,ab.

24. (Kiribati or Gilbert Islands or Phoenix Islands or Line Islands).ti,ab.

25. Laos/

26. (Laos or (Lao adj1 Democratic Republic)).ti,ab.

27. Micronesia/

28. Micronesia.ti,ab.

29. Mongolia/

30. Mongolia.ti,ab.

31. Myanmar/

32. (Myanmar or Burma).ti,ab.

33. Papua New Guinea/

34. (Papua New Guinea or German New Guinea or British New Guinea or Territory of Papua).ti,ab.

35. Philippines/

36. (Philippines or Philippine Islands).ti,ab.

37. "Independent State of Samoa"/

38. ((Samoa not American Samoa) or Western Samoa or Navigator Islands or Samoan Islands).ti,ab.

39. Solomon Islands.ti,ab.

40. Timor-Leste/

41. (Timor-Leste or East Timor or Portuguese Timor).ti,ab.

42. Vanuatu/

43. (Vanuatu or New Hebrides).ti,ab.

44. Vietnam/

45. (Viet Nam or Vietnam or French Indochina).ti,ab.

46. American Samoa/

47. American Samoa.ti,ab.

48. exp China/

49. China.ti,ab.

50. Fiji/

51. Fiji.ti,ab.

52. Malaysia/

53. (Malaysia or Malayan Union or Malaya).ti,ab.

54. Marshall Islands.ti,ab.

55. Nauru.ti,ab.

56. Thailand/

57. (Thailand or Siam).ti,ab.

58. Tonga/

59. Tonga.ti,ab.

60. (Tuvalu or Ellice Islands).ti,ab.

61. Kyrgyzstan/

62. (Kyrgyzstan or Kyrgyz Republic or Kirghizia or Kirghiz).ti,ab.

63. Tajikistan/

64. Tajikistan.ti,ab.

65. Ukraine/

66. Ukraine.ti,ab.

67. Uzbekistan/

68. Uzbekistan.ti,ab.

69. Albania/

70. Albania.ti,ab.

71. Armenia/

72. Armenia.ti,ab.

73. Azerbaijan/

74. Azerbaijan.ti,ab.

75. "Republic of Belarus"/

76. (Belarus or Byelarus or Byelorussia or Belorussia).ti,ab.

77. Bosnia-Herzegovina/

78. (Bosnia or Herzegovina).ti,ab.

79. Bulgaria/

80. Bulgaria.ti,ab.

81. "Georgia (Republic)"/

82. Georgia.ti,ab. not Georgia/

83. Kazakhstan/

84. (Kazakhstan or Kazakh).ti,ab.

85. Kosovo/

86. Kosovo.ti,ab.

87. Moldova/

88. Moldova.ti,ab.

89. Montenegro/

90. Montenegro.ti,ab.

91. "Republic of North Macedonia"/

92. North Macedonia.ti,ab.

93. Romania/

94. Romania.ti,ab.

95. exp Russia/

96. "Russia (Pre-1917)"/

97. USSR/

98. (Russia or Russian Federation or USSR or Union of Soviet Socialist Republics or Soviet Union).ti,ab.

99. Serbia/

100. Serbia.ti,ab.

101. Turkey/

102. (Turkey.ti,ab. not animal/) or (Anatolia or Asia Minor).ti,ab.

103. Turkmenistan/

104. Turkmenistan.ti,ab.

105. Belize/

106. (Belize or British Honduras).ti,ab.

107. Bolivia/

108. Bolivia.ti,ab.

109. El Salvador/

110. El Salvador.ti,ab.

111. Haiti/

112. (Haiti or Hayti).ti,ab.

113. Honduras/

114. Honduras.ti,ab.

115. Nicaragua/

116. Nicaragua.ti,ab.

117. Argentina/

118. (Argentina or Argentine Republic).ti,ab.

119. Brazil/

120. Brazil.ti,ab.

121. Colombia/

122. Colombia.ti,ab.

123. Costa Rica/

124. Costa Rica.ti,ab.

125. Cuba/

126. Cuba.ti,ab.

127. Dominica/

128. Dominica.ti,ab.

129. Dominican Republic/

130. Dominican Republic.ti,ab.

131. Ecuador/

132. Ecuador.ti,ab.

133. Grenada/

134. Grenada.ti,ab.

135. Guatemala/

136. Guatemala.ti,ab.

137. Guyana/

138. (Guyana or British Guiana).ti,ab.

139. Jamaica/

140. Jamaica.ti,ab.

141. Mexico/

142. (Mexico or United Mexican States).ti,ab.

143. Panama/

144. Panama.ti,ab.

145. Paraguay/

146. Paraguay.mp.

147. Peru/

148. Peru.ti,ab.

149. Saint Lucia/

150. (St Lucia or Saint Lucia or Iyonala or Hewanorra).ti,ab.

151. "Saint Vincent and the Grenadines"/

152. (Saint Vincent or St Vincent or Grenadines).ti,ab.

153. Suriname/

154. (Suriname or Dutch Guiana).ti,ab.

155. Venezuela/

156. Venezuela.ti,ab.

157. Algeria/

158. Algeria.ti,ab.

159. Djibouti/

160. (Djibouti or French Somaliland).ti,ab.

161. Egypt/

162. Egypt.ti,ab.

163. Iran/

164. (Iran or Persia).ti,ab.

165. Morocco/

166. Morocco.ti,ab.

167. Tunisia/

168. Tunisia.mp.

169. (Gaza or West Bank or Palestine).ti,ab.

170. Iraq/

171. (Iraq or Mesopotamia).ti,ab.

172. Jordan/

173. Jordan.ti,ab.

174. Lebanon/

175. (Lebanon or Lebanese Republic).ti,ab.

176. Libya/

177. Libya.ti,ab.

178. Afghanistan/

179. Afghanistan.ti,ab.

180. Bangladesh/

181. Bangladesh.ti,ab.

182. Bhutan/

183. Bhutan.ti,ab.

184. exp India/

185. India.ti,ab.

186. Nepal/

187. Nepal.ti,ab.

188. Pakistan/

189. Pakistan.ti,ab.

190. Sri Lanka/

191. (Sri Lanka or Ceylon).ti,ab.

192. Maldives.ti,ab. [UPPER MIDDLE INCOME COUNTRIES IN SOUTH ASIA]

193. Angola/

194. Angola.ti,ab.

195. Benin/

196. Benin.ti,ab.

197. Cameroon/

198. (Cameroon or Kamerun or Cameroun).ti,ab.

199. Cape Verde/

200. (Cape Verde or Cabo Verde).ti,ab.

201. Comoros/

202. (Comoros or Glorioso Islands or Mayotte).ti,ab.

203. Congo/

204. (Congo not ((Democratic Republic adj3 Congo) or congo red or crimean-congo)).ti,ab.

205. Cote d'Ivoire/

206. (Cote d'Ivoire or Cote dIvoire or Ivory Coast).ti,ab.

207. Eswatini/

208. (eSwatini or Swaziland).ti,ab.

209. Ghana/

210. (Ghana or Gold Coast).ti,ab.

211. Kenya/

212. (Kenya or East Africa Protectorate).ti,ab.

213. Lesotho/

214. (Lesotho or Basutoland).ti,ab.

215. Mauritania/

216. Mauritania.ti,ab.

217. Nigeria/

218. Nigeria.ti,ab.

219. (Sao Tome adj2 Principe).ti,ab.

220. Senegal/

221. Senegal.ti,ab.

222. Tanzania/

223. (Tanzania or Tanganyika or Zanzibar).ti,ab.

224. Zambia/

225. (Zambia or Northern Rhodesia).ti,ab.

226. Zimbabwe/

227. (Zimbabwe or Southern Rhodesia).ti,ab.

228. Botswana/

229. (Botswana or Bechuanaland or Kalahari).ti,ab.

230. Equatorial Guinea/

231. (Equatorial Guinea or Spanish Guinea).ti,ab.

232. Gabon/

233. (Gabon or Gabonese Republic).ti,ab.

234. Mauritius/

235. (Mauritius or Agalega Islands).ti,ab.

236. Namibia/

237. (Namibia or German South West Africa).ti,ab.

238. South Africa/

239. (South Africa or Cape Colony or British Bechuanaland or Boer Republics or Zululand or Transvaal or Natalia Republic or Orange Free State).ti,ab.

240. Syria/

241. (Syria or Syrian Arab Republic).ti,ab.

242. Yemen/

243. Yemen.ti,ab.

244. Burkina Faso/

245. (Burkina Faso or Burkina Fasso or Upper Volta).ti,ab.

246. Burundi/

247. (Burundi or Ruanda-Urundi).ti,ab.

248. Central African Republic/

249. (Central African Republic or Ubangi-Shari).ti,ab.

250. Chad/

251. Chad.ti,ab.

252. "Democratic Republic of the Congo"/

253. (((Democratic Republic or DR) adj2 Congo) or Congo-Kinshasa or Belgian Congo or Zaire or Congo Free State).ti,ab.

254. Eritrea/

255. Eritrea.ti,ab.

256. Ethiopia/

257. (Ethiopia or Abyssinia).ti,ab.

258. Gambia/

259. Gambia.ti,ab.

260. Guinea/

261. (Guinea not (New Guinea or Guinea Pig* or Guinea Fowl or Guinea-Bissau or Portuguese Guinea or Equatorial Guinea)).ti,ab.

262. Guinea-Bissau/

263. (Guinea-Bissau or Portuguese Guinea).ti,ab.

264. Liberia/

265. Liberia.ti,ab.

266. Madagascar/

267. (Madagascar or Malagasy Republic).ti,ab.

268. Malawi/

269. (Malawi or Nyasaland).ti,ab.

270. Mali/

271. Mali.ti,ab.

272. Mozambique/

273. (Mozambique or Mocambique or Portuguese East Africa).ti,ab.

274. Niger/

275. (Niger not (Aspergillus or Peptococcus or Schizothorax or Cruciferae or Gobius or Lasius or Agelastes or Melanosuchus or radish or Parastromateus or Orius or Apergillus or Parastromateus or Stomoxys)).ti,ab.

276. Rwanda/

277. (Rwanda or Ruanda).ti,ab.

278. Sierra Leone/

279. (Sierra Leone or Salone).ti,ab.

280. Somalia/

281. (Somalia or Somaliland).ti,ab.

282. South Sudan/

283. South Sudan.ti,ab.

284. Sudan/

285. Sudan.ti,ab.

286. Togo/

287. (Togo or Togolese Republic or Togoland).ti,ab.

288. Uganda/

289. Uganda.ti,ab.

290. (qualitative* or interview* or "focus group*" or "grounded theory" or phenomenology or phenomenologies or phenomenological or ethnography or ethnographies or ethnographic or ethnographical or story or stories or storytelling or "story-telling" or "story telling" or "open-ended" or "open ended" or "open question*" or "discourse analys*" or "discursive analys*" or "content analys*" or "thematic analys*" or "framework analys*").mp. [mp=title, book title, abstract, original title, name of substance word, subject heading word, floating sub-heading word, keyword heading word, organism supplementary concept word, protocol supplementary concept word, rare disease supplementary concept word, unique identifier, synonyms, population supplementary concept word, anatomy supplementary concept word]

291. blood pressure.mp. or exp Blood Pressure/

292. exp Hypertension, Pulmonary/ or exp Hypertension/ or exp Cardiovascular Diseases/ or hypertens*.mp. or exp Blood Pressure/

293. exp Univentricular Heart/ or exp Carcinoid Heart Disease/ or exp Heart Septum/ or exp Heart Bypass, Right/ or exp Heart Valve Prosthesis Implantation/ or exp American Heart Association/ or exp Heart Rate Determination/ or exp Heart Rate/ or exp Heart Rate, Fetal/ or exp Mitochondria, Heart/ or exp Heart Neoplasms/ or exp Heart Rupture/ or exp Heart Valves/ or exp Heart Valve Prosthesis/ or exp Heart Ventricles/ or exp Heart Failure, Diastolic/ or exp Heart Injuries/ or exp Heart Septal Defects, Ventricular/ or exp Heart Function Tests/ or exp Heart Valve Diseases/ or exp Heart Disease Risk Factors/ or exp Rheumatic Heart Disease/ or exp Fetal Heart/ or exp Heart Diseases/ or heart.mp. or exp Pulmonary Heart Disease/ or exp Heart Massage/ or exp Heart Sounds/ or exp Heart Septal Defects/ or exp Heart/ or exp Heart Transplantation/ or exp Heart Failure, Systolic/ or exp Hypoplastic Left Heart Syndrome/ or exp Heart Block/ or exp Heart Bypass, Left/ or exp Heart Auscultation/ or exp Heart Arrest/ or exp Heart Aneurysm/ or exp Heart Defects, Congenital/ or exp Isolated Heart Preparation/ or exp Heart Failure/ or exp Heart-Lung Machine/ or exp Heart-Lung Transplantation/ or exp "National Heart, Lung, and Blood Institute (U.S.)"/ or exp Heart Conduction System/ or exp Heart, Artificial/ or exp Heart Valve Prolapse/ or exp Heart-Assist Devices/ or exp Crisscross Heart/ or exp Heart Arrest, Induced/ or exp Heart Rupture, Post-Infarction/ or exp Heart Septal Defects, Atrial/ or exp Heart Murmurs/ or exp Heart Atria/

294. exp Granulomatous Disease, Chronic/ or exp Multiple Sclerosis, Chronic Progressive/ or exp Chronic Pain/ or exp Wasting Disease, Chronic/ or exp Candidiasis, Chronic Mucocutaneous/ or exp Leukemia, Lymphocytic, Chronic, B-Cell/ or exp Pancreatitis, Chronic/ or exp Bronchitis, Chronic/ or exp Leukemia, Myelogenous, Chronic, BCR-ABL Positive/ or exp Acute-On-Chronic Liver Failure/ or exp Hepatitis B, Chronic/ or exp Kidney Failure, Chronic/ or exp Polyradiculoneuropathy, Chronic Inflammatory Demyelinating/ or chronic*.mp. or exp Renal Insufficiency, Chronic/ or exp Chronic Limb-Threatening Ischemia/ or exp "Chemical and Drug Induced Liver Injury, Chronic"/ or exp Chronic Urticaria/ or exp Hematoma, Subdural, Chronic/ or exp Hepatitis C, Chronic/ or exp Hepatitis D, Chronic/ or exp Multiple Chronic Conditions/ or exp Chronic Periodontitis/ or exp Chronic Disease/ or exp Pulmonary Disease, Chronic Obstructive/ or exp Fatigue Syndrome, Chronic/ or exp Leukemia, Myelomonocytic, Chronic/ or exp "Chronic Kidney Disease-Mineral and Bone Disorder"/

295. exp Diabetes Mellitus, Experimental/ or exp Diabetes Mellitus, Type 2/ or exp Diabetes Mellitus, Type 1/ or diabet*.mp. or exp Blood Glucose/ or exp Diabetes Mellitus/ or exp Diabetic Nephropathies/

296. ((HIV* or AIDS* or "acquired immunodeficiency syndrome" or "acquired immune-deficiency syndrome" or "acquired-immunodeficiency-syndrome" or "human immunodeficiency virus" or "human immune-deficiency virus" or "human-immunodeficiency-virus") adj3 (people* or person* or patient* or positive*)).mp. [mp=title, book title, abstract, original title, name of substance word, subject heading word, floating sub-heading word, keyword heading word, organism supplementary concept word, protocol supplementary concept word, rare disease supplementary concept word, unique identifier, synonyms, population supplementary concept word, anatomy supplementary concept word]

297. 291 or 292 or 293 or 294 or 295 or 296

298. ("USA" or "U.S.A" or "U.S." or "United States of America" or "African American*" or "Latin American*" or "Alabama" or "Alaska" or "Arizona" or "Arkansas" or "California" or "Colorado" or "Connecticut" or "Delaware" or "Florida" or "Georgia" or "Hawaii" or "Idaho" or "Illinois" or "Indiana" or "Iowa" or "Kansas" or "Kentucky" or "Louisiana" or "Maine" or "Maryland" or "Massachusetts" or "Michigan" or "Minnesota" or "Mississippi" or "Missouri" or "Montana" or "Nebraska" or "Nevada" or "New Hampshire" or "New Jersey" or "New Mexico" or "New York" or "North Carolina" or "North Dakota" or "Ohio" or "Oklahoma" or "Oregon" or "Pennsylvania" or "Rhode Island" or "South Carolina" or "South Dakota" or "Tennessee" or "Texas" or "Utah" or "Vermont" or "Virginia" or "Washington" or "West Virginia" or "Wisconsin" or "Wyoming").ab,ti.

299. or/3-289

300. 1 and 2 and 290 and 297 and 299

301. 300 not 298

302. limit 301 to english language

303. limit 302 to journal article
